# Huge desmoid tumor of the anterior abdominal wall mimicking an intraabdominal mass in a postpartum woman: a case report

**DOI:** 10.11604/pamj.2013.14.52.2414

**Published:** 2013-02-07

**Authors:** Khaled Trigui, Mahdi Bouassida, Houda Kilani, Mohamed Mongi Mighri, Selim Sassi, Fathi Chebbi, Hassen Touinsi, Sadok Sassi

**Affiliations:** 1 Department of surgery, MTM Hospital, Nabeul, Tunisia; 2 Department of cytology, MTM Hospital, Nabeul, Tunisia

**Keywords:** Desmoid tumors, post partum, surgery

## Abstract

Desmoid tumors are benign neoplasms that most often arise from muscle aponeurosis and have been associated with both trauma and pregnancy. The etiology of desmoids has not been determined. We report the case of anterior abdominal wall desmoid tumor in a female patient with previous history of cesarean section. Preoperative ultrasound and computed tomography demonstrated a large mass mimicking a large hematoma or an intraabdominal mass. The tumor was removed by wide excision with safe margins. The abdominal wall defect was reconstructed with polypropylene mesh. Subsequent histology revealed a desmoid tumor. Desmoid tumors in females are often associated with pregnancy or occur post-partum. The reasons behind this association are unclear. The most common sites are in the abdominal muscles.

## Introduction

Desmoid tumors are rare neoplasms, accounting for 0.3% of all neoplasms and less than 3% of all soft tissue tumors with an estimated incidence in the general population of 2-4 per million of population per year [1–3]. They are histologically benign arising from connective tissue of muscles, the fascia, or the aponeurosis and may occur at multiple anatomic sites [4]. The term “desmoids”, coined by Muller in 1838, was based on the Greek etymology “Desmos”, which means tendonlike. Pregnancy associated desmoids tumors have been a subject of interest since the first description by Macfarlene in 1832 [5].

## Patient and observation

A 28 years old woman consulted for a firm mass in the left upper abdomen. The interview found no history of family or personal neoplasms, familial adenomatous polyposis (FAP), colorectal disease, no concept of hormonal contraception, alcoholism or smoking. The patient was secondparous delivering by cesarean section in her first pregnancy five years earlier, through a Pfannestiel incision and by vaginal delivery in her second pregnancy 16 months ago.

The patient noticed a small tumor in the anterior abdominal wall for the first time 6 months post partum. This tumor, initially neglected by the patient, was gradually increasing in size and associated with a sustained pressure in her cesarean scar inciting the woman for consulting 10 months after its discovery.

On clinical examination, the general condition was maintained. We found a huge anterior abdominal wall mass measuring 20 cm × 25 cm, firm, painless, fixed to the superficial and deep levels. Blood parameters analyses were within normal range.

**Figure 1:**
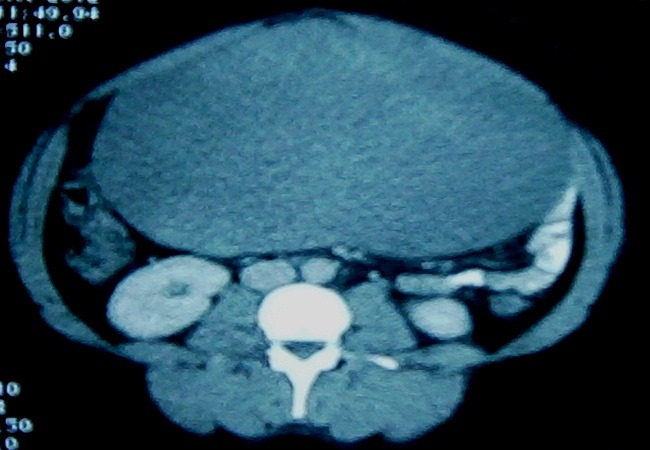
Pelvi-abdominal computed tomography showing a large, well-defined, oval mass measuring 23 × 18 × 13 cm in dimensions overlapping the left side of the abdominal cavity

Ultrasonography showed an oval mass of 21 centimeters with hypo and heterogeneous echogenicity and well defined margins in the left anterolateral abdominal wall. Preoperative computed tomography scan images revealed a well-circumscribed, large mass (23 × 18 × 13 cm) with heterogeneous density. The mass was close to the left rectus abdominal muscle and after intravenous administration of contrast medium it demonstrated no enhancement even in the delayed images (Figure 1). After these both investigations, the mass was thought to be a hematoma.

**Figure 2:**
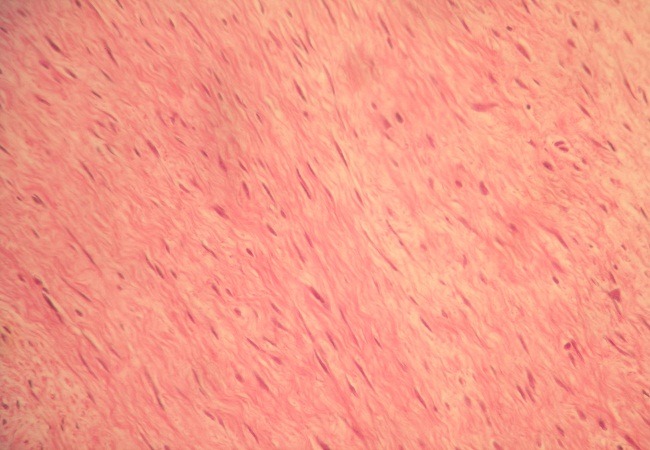
Histopathologic findings: spindle cells of low cell-density with few mitoses (X10)

The decision was made for laparotomy, which was performed via a midline incision. A large mass attached firmly to the inner aspect of the abdominal wall was found intraoperatively with no infiltration of abdominal cavity organs. Surgeons took over and the mass was removed by wide excision with a safe margins. The defect was reconstructed with polypropylene mesh. Subsequent histopathology revealed features of a desmoid tumor (Figure 2).

The postoperative course was uneventful and the patient was discharged at the 9th postoperative day. Coloscopy, performed 3 months later, was normal.

## Discussion

Desmoid tumors can occur sporadically or as part of an inherited syndrome, familial adenomatous polyposis [6]. There is a clear link between the development of desmoids tumors, pregnancy, and the postpartum state. This association has been noted many times in the past, mainly in single case reports in the literature [7–9].Several explanations were suggested having in common the physical, hormonal, and immunologic changes observed during the pregnancy or the post partum period. The most important of these explanations are probably hormonal factors. This suggestion is strengthened by reports of spontaneous regression of pregnancy associated desmoids after delivery and without treatment.

Furthermore authors reports that some desmoids, both in pregnant and non-pregnant patients, respond to treatment with the anti-estrogen tamoxifen [10]. What is most interesting about most of previous cases, and this reported here is that the desmoids arising in this situation are almost always in the abdominal wall, abdominal scars and trauma including those resulting of stretching and/or tearing of the aponeurosis in the abdominal muscles due to the pregnancy state were evoqued to be an important factor in generating such type of neoplasms, although in a few cases the tumor arose elsewhere in the body [11–14].

The etiologic diagnosis for an abdominal mass is varied especially in females. In fact, these masses may arise from the reproductive organs, gastrointestinal system, abdominal wall, urinary system, adjacent soft tissues, and metastases. Previous case reports showed that desmoids tumors may mimic other intra abdominal tumors [15]. This case report shows that some abdominal tumors may be clinically challenging for clinicians. The confusion in diagnosis may result from unusual locations, large-sized tumors, and discordant or insufficient results obtained using different diagnostic tools.

In many times, the diagnosis is suspected intraoperatively, showing a tumor growing from the abdominal wall. Most often they are confined locally, but may show infiltration of adjacent structures. Histology is the only evidentiary method which demonstrates long fascicles of spindle cells of variable cell-density with few mitoses and absence of atypical nucleus-separations. Characteristically, there is a diffuse cell infiltration of adjacent tissue structures. Immunohistochemical response for actin can be partially positive and immunohistochemical muscle cell markers delimit desmoid tumors from fibro sarcomas [16]. Surgery remains the mainstay of treatment in all patients with extra-abdominal desmoid tumors. The literature presents conflicting data concerning the importance of complete resection. Some authors report that recurrence is independent of negative margins [17, 18] whereas, others demonstrate higher local recurrence rates after close or positive margins, and recommend aggressive resection. In addition to surgery other therapies such as anti-estrogens, chemotherapy, and radiation have been proposed [19], however, the numbers of patients treated with these agents have been small, the durability of the responses has been poorly documented, and the response rates have not approached those of conventional surgery. Therefore, the use of such treatments remains experimental or applicable to situations in which the more conventional modalities have already been tried.

## Conclusion

Desmoid tumors in females are often associated with pregnancy or occur post-partum. The reasons behind this association are unclear. The most common sites are in the abdominal muscles, but they have been reported in multiple other areas of the body. Surgical resection is the treatment of choice and is usually curative even when surgical margins are involved.
